# Impact of Whole-Body Cryostimulation (WBC) on Hemostatic Parameters in Patients with Multiple Sclerosis

**DOI:** 10.3390/jcm15166425

**Published:** 2026-08-20

**Authors:** Ewa Zielińska-Nowak, Angela Dziedzic, Anna Lipert, Joanna Kolodziejczyk-Czepas, Joanna Saluk, Elżbieta Miller

**Affiliations:** 1 Department of Neurological Rehabilitation, Medical University of Lodz, Milionowa 14, 90-001 Lodz, Poland; elzbieta.dorota.miller@umed.lodz.pl; 2 Department of General Biochemistry, Faculty of Biology and Environmental Protection, University of Lodz, Pomorska 141/143, 90-236 Lodz, Poland; angela.dziedzic@biol.uni.lodz.pl (A.D.); joanna.kolodziejczyk@biol.uni.lodz.pl (J.K.-C.); joanna.saluk@biol.uni.lodz.pl (J.S.); 3 Cardiovascular and Metabolic Diseases Prevention Laboratory, Department of Preventive Medicine, Medical University of Lodz, 92-213 Lodz, Poland; anna.lipert@umed.lodz.pl

**Keywords:** multiple sclerosis, whole-body cryostimulation, coagulation, fibrinolysis, hemostasis

## Abstract

**Background:** Multiple sclerosis (MS) involves neuroinflammation, demyelination and hemostatic disturbances linked to blood–brain barrier (BBB) disruption and the resulting neurovascular dysfunction. Whole-body cryostimulation (WBC) is used as an adjunct therapy; however, its influence on coagulation and fibrinolysis remains unclear. This study evaluated the hemostatic effects of a 10-session WBC protocol in MS and non-MS participants. **Methods:** Seventy-two adults (MS *n* = 29; non-MS *n* = 43) underwent 10 consecutive WBC sessions (3 min, −120 °C to −130 °C). Blood samples were collected at baseline (T1), after the 10th session (T2), and 10 days later (T3). Hemostatic parameters were evaluated using plasma coagulation times (PT, TT, aPTT), as well as thrombus formation under flow conditions (T-TAS^®^) and Clot Formation and Fibrinolysis (CFF). **Results**: WBC induced mild alterations in coagulation times, particularly within the extrinsic (PT) and final fibrin-generation (TT) pathways, without affecting the intrinsic pathway (aPTT). No significant changes in thrombus formation or fibrinolysis were detected in T-TAS^®^ or CFF methods, suggesting preserved overall hemostatic balance. **Conclusions:** In both groups, a 10-session WBC protocol did not disrupt global hemostatic function, despite minor changes in several coagulation parameters. These findings support the hemostatic safety of WBC and indicate that the intervention does not promote a prothrombotic state in patients with MS.

## 1. Introduction

Multiple sclerosis (MS) is a chronic inflammatory demyelinating disease of the central nervous system (CNS), driven by an autoimmune mechanism and characterized by chronic neuroinflammation, demyelination, and progressive neurodegeneration [1,2]. Growing evidence indicates that MS extends beyond an immune-mediated disorder and encompasses substantial neurovascular dysfunction, including endothelial impairment, disrupted blood–brain barrier (BBB) integrity, altered cerebral perfusion and dysregulated hemostasis [3,4,5,6]. Hemostatic abnormalities in MS are increasingly recognized not only as secondary markers of systemic inflammation but also as potential contributors to disease propagation, linking thrombosis, neuroinflammation, and autoimmunity [7]. Perturbations within the coagulation and fibrinolytic systems, ranging from altered platelet activity to fibrin dynamics and impaired fibrinolysis, have been documented in MS. Because endothelial function is closely linked to the regulation of coagulation and fibrinolysis, the neurovascular and endothelial abnormalities observed in MS may also be associated with disturbances in clot initiation, fibrin formation, clot stabilization, and lysis [8,9,10,11]. Accordingly, dynamic plasma-based assays may complement conventional clotting times by characterizing the kinetics of clot formation and fibrinolysis [12]. Clinically, individuals with MS exhibit an approximately a twofold higher risk of venous thromboembolism compared with the general population [13] underscoring the relevance of hemostatic dysregulation in a disease not traditionally classified as prothrombotic. Nevertheless, the mechanistic interactions between coagulation pathways, BBB injury, neuroinflammation, and disease progression remain insufficiently understood, and therapeutic modulation of coagulation in MS remains largely unexplored.

Whole-body cryostimulation (WBC), a brief exposure to extremely low temperatures (typically −110 °C to −160 °C for 2–3 min), has gained popularity as an adjunctive intervention in inflammatory and autoimmune conditions, including MS [14,15,16]. WBC exerts systemic anti-inflammatory, antioxidant, neuromodulatory, and vascular effects, mimicking certain physiological responses observed after moderate exercise [17]. Early studies in MS indicate improvements in fatigue, mood, physical functioning, and markers of oxidative stress following repeated WBC sessions [16,17,18,19,20]. In consideration of the emerging concept that neuroinflammation, BBB disruption, oxidative stress, and endothelial dysfunction act synergistically in MS pathogenesis, WBC represents a plausible multimodal intervention targeting several disease-relevant pathways simultaneously [21].

Current data from non-neurological populations are limited and mixed; in rheumatoid arthritis, WBC has been shown to induce transient hemostatic and fibrinolytic shifts, raising questions about its impact in individuals with pre-existing prothrombotic tendencies [22]. Considering the heightened thrombotic risk reported in MS and the growing clinical use of WBC, a careful evaluation of its effects on coagulation and fibrinolysis in this population is warranted. Elucidating whether WBC can modulate hemostatic function in MS is not only novel from a pathophysiological standpoint but also carries potential clinical significance. It may not only provide novel mechanistic insights but also suggest potential additional therapeutic benefits of cryotherapy in mitigating thrombotic risk and inflammation-driven coagulopathies in this population. Demonstrating a neutral or favorable effect would support the safety of WBC in MS, while evidence of procoagulant activity would prompt caution and mechanistic investigation. Taking into account the multifaceted pathophysiology of MS, which is characterized by interactions among the immune, nervous and vascular systems, as well as the therapeutic benefits of WBC administration in patients with MS, the aim of this study is to investigate the safety of using WBC in MS patients by assessing the hemostatic response to WBC and to determine whether this response differs from that observed in neurologically healthy participants. Moreover, this is the first study to evaluate global thrombotic potential in response to WBC in patients with MS, extending the assessment beyond conventional coagulation tests by capturing dynamic thrombus formation and thereby providing a more comprehensive evaluation of hemostatic safety in this population.

## 2. Materials and Methods

### 2.1. Participants

**Figure 1:**
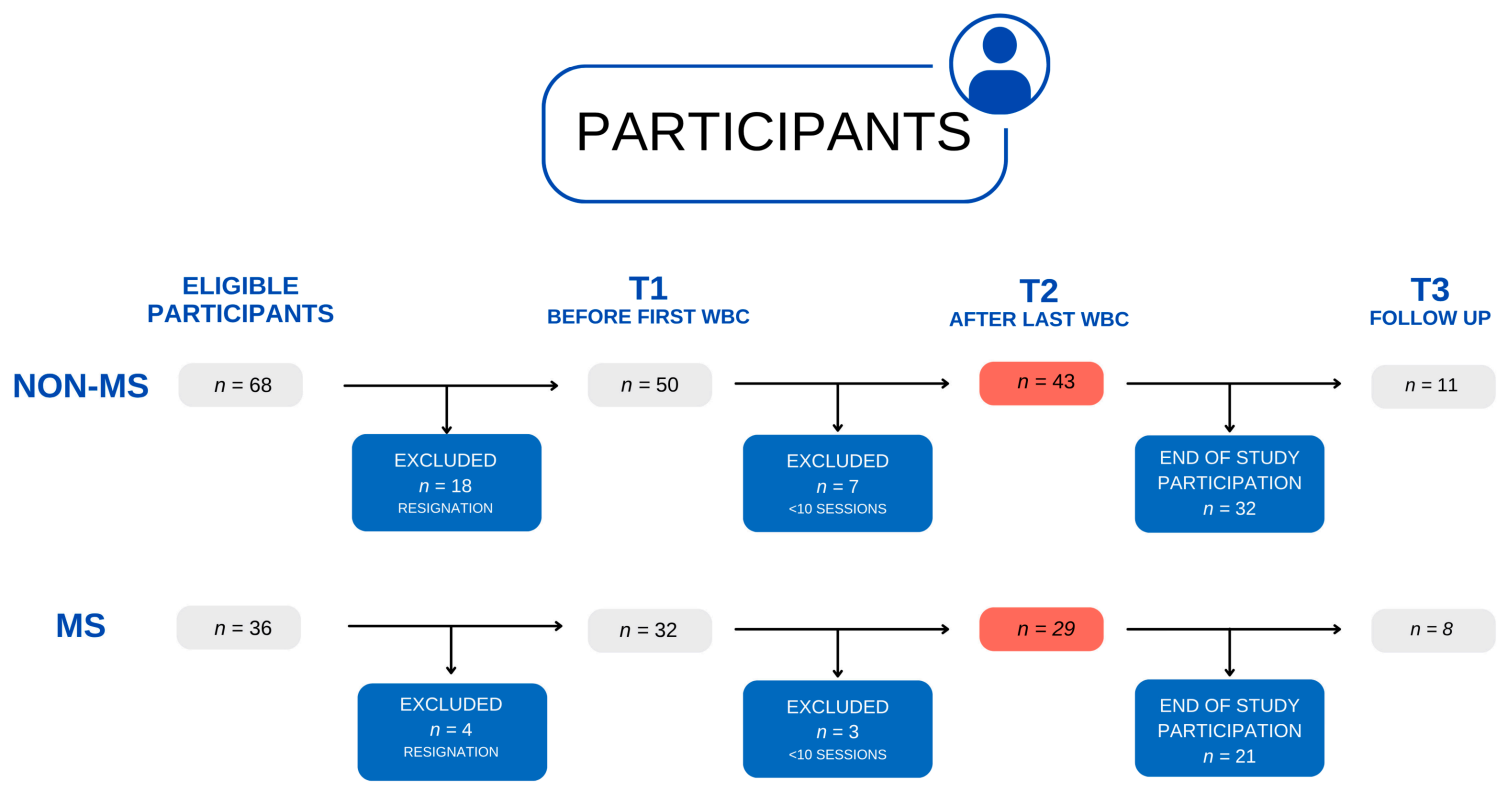
Participant flowchart. The orange circles indicate the number of participants who were assessed after 10 WBC sessions.

This study was conducted at the Creator Rehabilitation Center in Łódź, Poland, and involved 72 adults referred for WBC. Participants were categorized into two groups: 43 individuals without neurological disease (non-MS group, comprising patients with degenerative joint disease such as osteoarthritis) and 29 patients with a confirmed diagnosis of MS (MS group) (Figure 1).

Recruitment was conducted sequentially, with the non-MS group recruited first, followed by the MS group. Similar group sizes were initially planned. However, recruitment of eligible participants with MS proved considerably more difficult because fewer patients were referred for WBC and many did not meet the eligibility criteria.

All patients with MS had an established diagnosis confirmed by a neurologist and were under regular neurological care. At the time of the study, about 90% were receiving disease-modifying therapies (DMTs). These included ofatumumab (*n* = 7), dimethyl fumarate (*n* = 6), natalizumab (*n* = 3), ozanimod (*n* = 2), glatiramer acetate (*n* = 1), interferon beta-1a (*n* = 1), interferon beta-1b (*n* = 1), cladribine (*n* = 1), and ocrelizumab (*n* = 1). In three treated participants, the specific DMT was not recorded, whereas three participants were not receiving DMTs. No participants were receiving systemic corticosteroids or non-steroidal anti-inflammatory drugs. Most participants had relapsing–remitting MS (RRMS, ≈76%), followed by primary progressive MS (PPMS, ≈14%) and undetermined disease course (≈10%). Their disability status was generally mild. The average Expanded Disability Status Scale (EDSS) score was 2.6, with over 80% scoring below 4. Only one patient used a walking aid (EDSS = 6). Most non-MS participants were diagnosed with musculoskeletal conditions (primarily knee or spine osteoarthritis) and had no known neurological disorders.

Inclusion criteria for both groups were age between 18 and 85 years, both sexes, a physician’s referral for 10 WBC sessions, no contraindications to WBC, and the ability to consent and comply with the study protocol (including post-WBC exercise). For the MS group, an additional criterion was an EDSS score ≤ 6.0 to ensure that participants could safely complete all assessments. Exclusion criteria included any serious health conditions that would contraindicate WBC exposure, such as uncontrolled hypertension, recent cardiovascular events (e.g., myocardial infarction within 6 months), severe heart or lung disease, a history of deep vein thrombosis or other thromboembolic events, seizure disorders, active infections or fever, severe anemia, and cold intolerance (e.g., cryoglobulinemia or Raynaud’s disease). Participants undergoing intensive physiotherapy or experiencing significant changes in lifestyle or pharmacotherapy during the study period were excluded. Current use of anticoagulant or antiplatelet medication was also an exclusion criterion, and participants were advised to avoid supplements or herbal products that could affect coagulation or hemostasis throughout the study. All participants were screened for eligibility by a physician before enrollment to ensure patient safety.

The study was approved by the Ethics Committee of the Medical University of Łódź, Poland (approval no. RNN/97/23/KE, date of approval: 18 April 2023), and all procedures were performed in accordance with the Declaration of Helsinki. Each participant provided written informed consent prior to inclusion in the research.

### 2.2. Study Design

Participants underwent a standardized WBC intervention consisting of 10 sessions administered over a two-week period (five sessions per week on weekdays, with no sessions on weekends). Each WBC session was conducted in a two-chamber cryogenic unit cooled with liquid nitrogen. The procedure for a single session was as follows: the participant first spent 30 s in a pre-chamber maintained at approximately −60 °C to allow adaptation, then immediately entered the main chamber for 2 min. The main chamber was set to −120 °C on the first day and the temperature was progressively lowered by 2 °C every other session, reaching −130 °C by the final (10th) session. After the 2-min exposure in the main chamber, the participant returned to the pre-chamber (−60 °C) for an additional ~30 s before exiting. Thus, the total cold exposure time per session was around 3 min. During each session, participants wore minimal clothing along with protective gear including insulated gloves, knee-high socks, wooden clogs, a woolen headband or cap covering the ears, and a surgical mask over the mouth and nose. Immediately upon exiting the cryochamber, each participant engaged in a light-intensity exercise session for 30 min under supervision. This post-WBC exercise (usually cycling on a stationary bicycle or performing gentle calisthenics) was intended to promote gradual rewarming and to augment the therapeutic effects of cryotherapy by stimulating circulation and muscle activity.

### 2.3. Assessment Protocol

**Figure 2:**
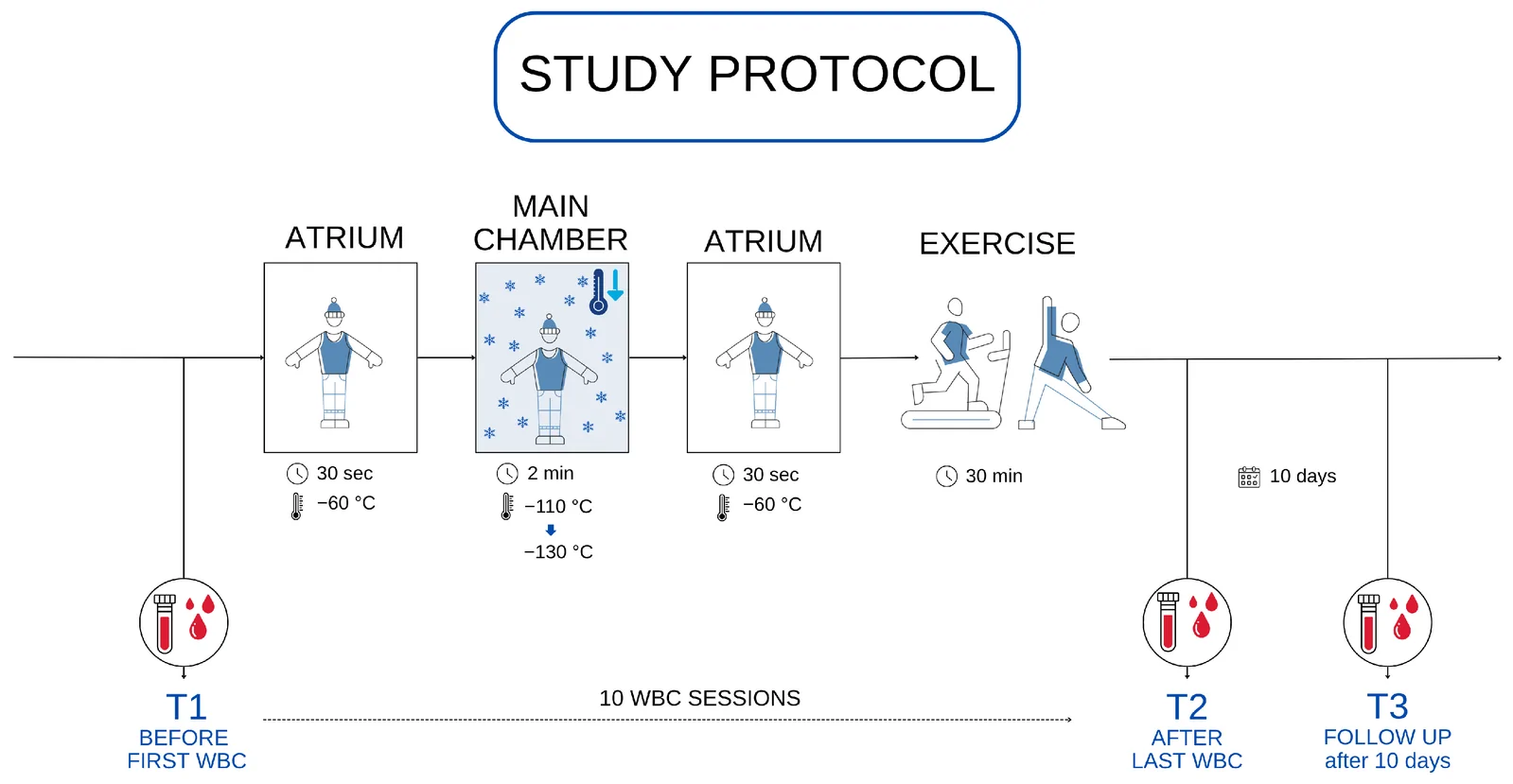
Study protocol.

Participants were evaluated at three time points relative to the WBC intervention: before the first session (T1, baseline), after completing the 10th session (T2, end of treatment), and 10 days after the last session (T3, follow-up). At each time point, blood samples were collected, and a series of laboratory and clinical assessments were performed as detailed below (Figure 2).

#### 2.3.1. Blood Sample Collection and Processing

Venous blood (approximately 10 mL) was drawn from the antecubital vein by a certified nurse at T1, T2, and T3 into sterile vacuum tubes containing 3.2% sodium citrate as an anticoagulant (S-Monovette^®^, Sarstedt, Nümbrecht, Germany). Samples were transported within one hour under controlled conditions and processed immediately to avoid artifactual platelet activation. Whole blood was first used to assess total clot formation under semi-physiological conditions, and was then centrifuged (15 min at 2000× *g*) to obtain platelet-poor plasma (PPP). The PPP was used for plasma-based hemostatic assays (coagulation times, thrombus formation, and clot dynamics). All sample handling and processing steps were standardized to ensure consistency across time points and participants.

#### 2.3.2. Total Thrombus-Formation Analysis System (T-TAS^®^)

The global thrombotic potential in response to WBC was evaluated using T-TAS^®^ technology. This method employs an AR-chip, a microfluidic chamber simulating arterial shear under in vitro condition, to provide an integrative measurement of platelet adhesion and activation, fibrin generation, and thrombus growth under the pressure flow. T-TAS analysis was performed using the AR-Chip on the Total Thrombus-formation Analysis System (T-TAS^®^ 01; Fujimori Kogyo Co. Ltd., ZACROS, Tokyo, Japan) in accordance with the manufacturer’s protocol. Citrate-anticoagulated whole blood was recalcified immediately before analysis by adding 20 µL of CaCTI reagent, containing calcium chloride and corn trypsin inhibitor (a specific factor XIIa inhibitor), to initiate the coagulation cascade. The microcapillaries of the AR-Chip are coated with collagen and tissue thromboplastin, enabling the assessment of fibrin-rich thrombus formation under a wall shear rate of 600 s^−1^. Thrombus growth was monitored in real time by a pressure sensor that recorded flow resistance due to occlusion. The results were presented as the area under the pressure–time curve (AUC).

#### 2.3.3. Coagulation Times

Changes between time points (T2–T1 and T3–T1) were compared separately in the MS and non-MS groups. Coagulation times, including prothrombin time (PT), thrombin time (TT) and activated partial thromboplastin time (aPTT), enable the detection of subtle alterations within distinct components of the coagulation cascade. PT reflects the function of the extrinsic pathway, which primarily depends on tissue factor and factor VII activity. Its standardized derivative, the International Normalized Ratio (INR), is calculated to account for inter-laboratory variability in thromboplastin sensitivity using the formula: INR = (Patient PT/Control PT)^ISI^, where ISI (International Sensitivity Index) denotes the responsiveness of the reagent applied. aPTT evaluates the intrinsic pathway, whereas TT reflects the final conversion of fibrinogen to fibrin under the influence of thrombin. By analysing these parameters, it is possible to distinguish pathway-specific responses to WBC and compare hemostatic profiles between MS and non-MS participants. PT, TT, apTT were measured using a semi-automated coagulometer (K-3002 Optic, KSELMED, Grudziądz, Poland) equipped with a thermostated module set to 37 °C, while reagents were purchased from DIAGON Kft. (Budapest, Hungary). For PT determination, 50 µL of plasma was pipetted into cuvettes, followed by the immediate addition of 100 µL of Dia-PT reagent. In the aPTT assay, 50 µL of plasma was incubated with 50 µL of Dia-APTT reagent for 180 s at 37 °C, after which 50 µL of Dia-CaCl_2_ was added to initiate clotting. For TT measurements, 100 µL of plasma was combined with 100 µL of DiaTT reagent, and clotting was monitored immediately. All procedures were performed according to standardized protocols to ensure consistency across samples.

#### 2.3.4. Clot Formation and Fibrinolysis (CFF) Assay

**Figure 3:**
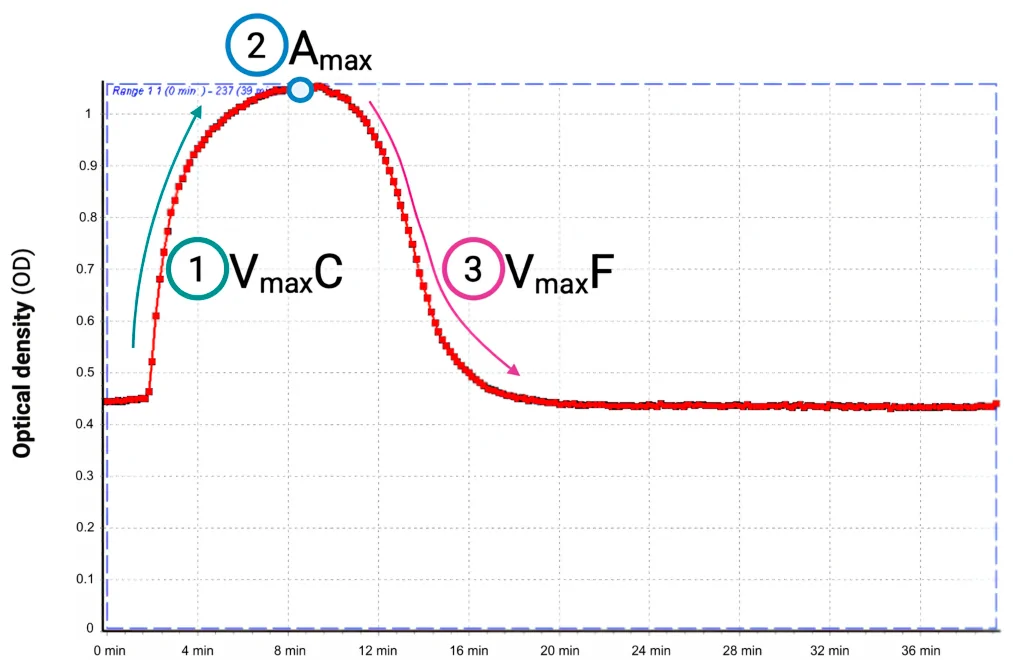
A representative curve illustrating the dynamic of clot formation and fibrinolysis using CFF assay. The assay begins with the activation of the plasma coagulation cascade, leading to fibrin clot formation (1), which is characterized by the maximum velocity of fibrin polymerization (V_maxC_). The peak of maximum absorbance (A_max_) corresponds to the fibrin stabilization phase and reflects the thickness of the formed clot (2). In the final phase (3), fibrinolytic mechanisms are activated, resulting in clot degradation, which is described by the maximum velocity of fibrinolysis (V_maxF_).

Thrombin was obtained from BioMed (Lublin, Poland), and tissue-type plasminogen activator (tPA) (Actilyse^®^) was obtained from Boehringer Ingelheim (Ingelheim am Rhein, Germany). Briefly, 100 µL of plasma was transferred to microplate wells, and 200 µL of a reagent mixture containing 0.75 U/mL thrombin, 225 ng/mL t-PA, and 7.5 mM CaCl_2_ in 0.05 M Tris-buffered saline (TBS) (0.9% NaCl, pH 7.4) was added. Absorbance at 360 nm was monitored kinetically for 60 min at 37 °C. The analysis focused on three parameters: the maximum velocity of fibrin polymerization (V_maxC_), indicating coagulation efficiency; maximum absorbance (A_max_), reflecting clot density and stability; and maximum velocity of clot lysis (V_maxF_), representing fibrinolytic activity. Kinetic analyses were performed using a SpectrostarNano microplate spectrophotometer (BMG LabTech, Ortenberg, Germany). A representative curve illustrating the kinetics of clot formation and fibrinolysis is presented in Figure 3.

### 2.4. Statistical Analysis

All statistical analyses were performed using Statistica software version 13.1 (StatSoft Inc., Tulsa, OK, USA). Prior to hypothesis testing, the distributions of continuous data were evaluated for normality using the Shapiro–Wilk test. Because many of the outcome variables deviated from a normal distribution, non-parametric statistical tests were primarily applied. The statistical analysis was performed using the Mann–Whitney U test for two independent groups, and Kruskal–Wallis ANOVA was used for more than two independent variables. To compare dependent variables, such as data across the intervention time points (within-group changes from T1 to T2, T1 to T3, etc.), the Friedman’s ANOVA with Kendall’s coefficient was applied, followed by the Wilcoxon signed-rank test. The effect size, measuring the differences within and between groups, was determined by Cohen’s d. Effect sizes were recorded as small (d = 0.2), medium (d = 0.5), and large (d = 0.8). Spearman’s correlation coefficient was used to assess the relationship between the variables. A two-tailed *p*-value < 0.05 was considered to indicate statistical significance for all tests. Data are presented with appropriate summary statistics (the median and 95% confidence intervals (CI) for non-parametric data or the mean ± standard deviation for approximately normally distributed data) as needed. All analyses were conducted using a complete-case approach (excluding the one MS participant with missing blood data), and no imputation was performed for missing values.

## 3. Results

A total of 72 participants completed the study at T2, of whom 20 agreed to participate in the follow-up evaluation (T3). No adverse events occurred during the study. The baseline demographics and clinical characteristics of both groups are presented in Table 1.

### 3.1. Assessment of Thrombus Formation Capacity Using the T-TAS^®^ System Following WBC in MS and Non-MS Participants

The key output parameter, the area under the curve (AUC), reflects cumulative thrombotic activity over time, initiated via the tissue factor pathway [23]. As presented in Table 2, no statistically significant changes in AUC values were observed in either group following WBC (Table 2).

**Figure 4:**
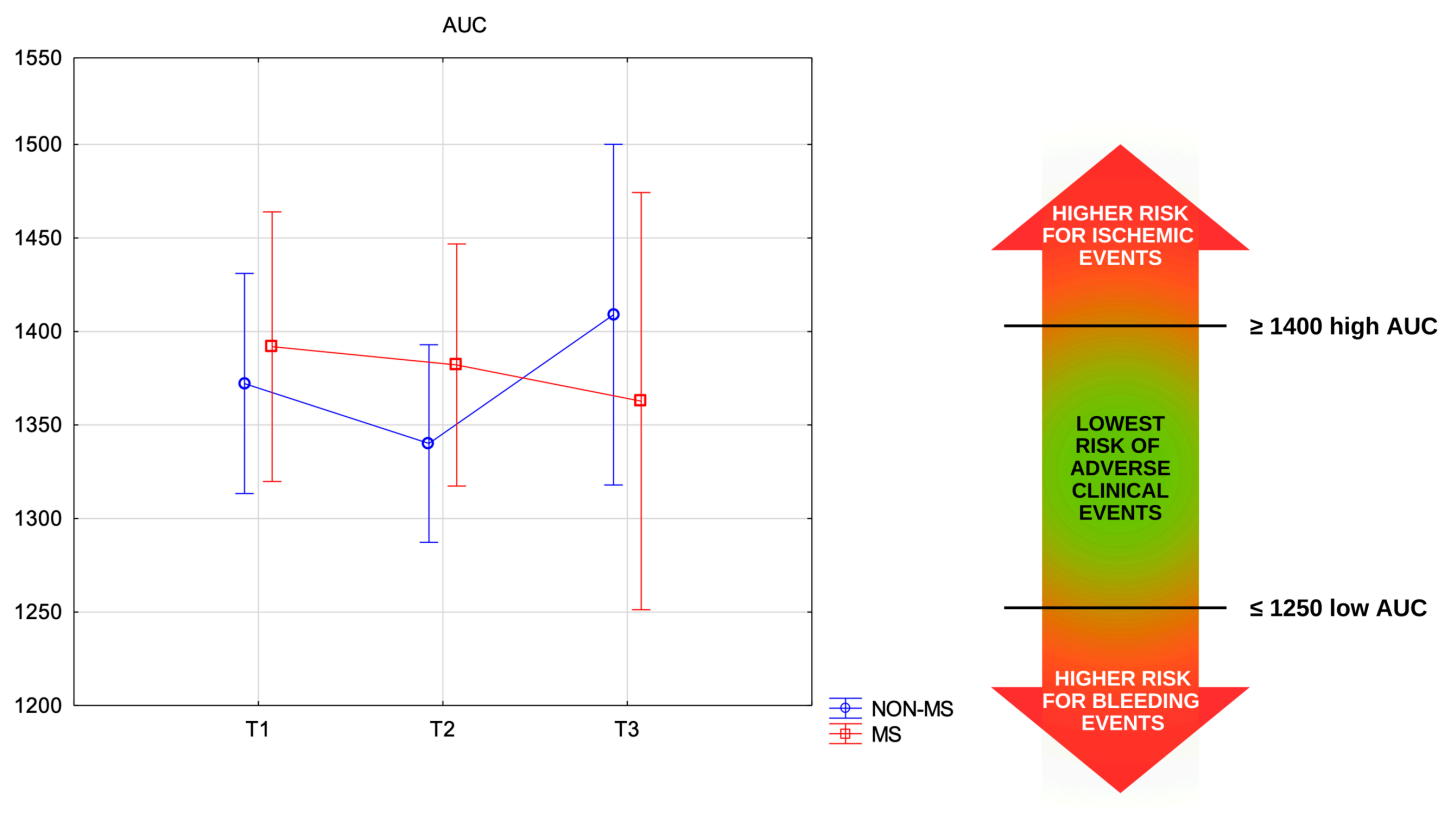
AUC parameter measured by T-TAS^®^ in MS and non-MS groups across three time points (T1, T2, T3). The graph illustrates the median AUC values accompanied by 95% confidence intervals (CI) (lower; upper limit) reflecting thrombus formation capacity in the non-MS (blue markers) and MS (red markers) groups at baseline (T1), immediately after 10 sessions of whole-body cryostimulation (T2), and at follow-up (T3, 10 days post-treatment). The reference range is visually indicated by the green horizontal band between 1250 and 1400 AUC units, which represents the zone of lowest risk for adverse clinical events. AUC values above 1400 suggest a higher thrombotic potential and increased risk of ischemic events, whereas values below 1250 are associated with a higher bleeding risk.

In the non-MS group, a non-significant decrease in AUC was observed immediately after treatment (Δ Changes: *p* = 0.133, d = 0.20) followed by a non-significant increase at follow-up (Δ Follow-up: *p* = 0.463, d = 0.61), slightly exceeding the 1400 threshold. By contrast, the MS group demonstrated a sustained decrease in AUC (Δ Changes: *p* = 0.755, d = 0.05), showing a slight downward trend within physiological range at T3 as well (Δ Follow-up: *p* = 0.499, d = 1.22). Importantly, in both groups AUC values remained largely within the recommended “low-risk” therapeutic window (≈1250–1400), corresponding to approximate values provided by the manufacturer, and is broadly consistent with normal ranges reported elsewhere [24,25]. Non-MS participants temporarily approached the high-AUC threshold (≥1400), whereas MS participants showed progressive decrease (Figure 4).

A substantial effect size was identified for the MS group for the change between T3 and T1 (d = 1.22), although this did not reach statistical significance (*p* = 0.499). This may indicate a potentially meaningful change that was not statistically significant possibly due to the limited sample size. Additionally, a moderate effect size was observed in the non-MS group in the change between T3 and T1 (d = 0.61), although this result was also not statistically significant.

### 3.2. Changes in Coagulation Times (PT, TT and aPTT) Following WBC in MS and Non-MS Participants

To assess the impact of WBC on different components of the coagulation cascade, standard plasma clotting times were measured at baseline (T1), immediately after the 10-session intervention (T2), and after a 10-day follow-up period (T3). All changes in coagulation times are presented in Table 3.

**Figure 5:**
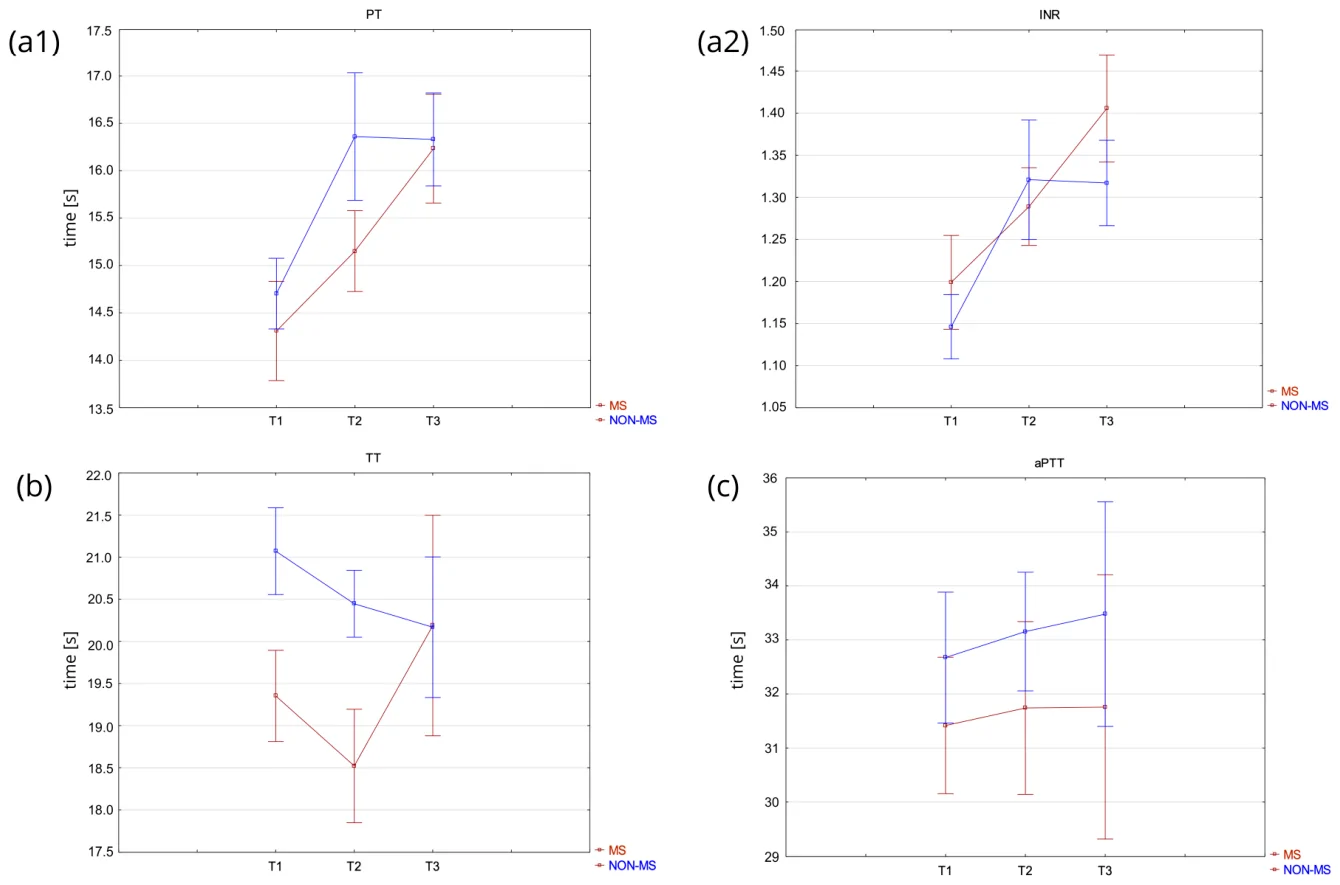
Changes in plasma coagulation parameters across three study time points (T1—baseline, T2—after 10 WBC sessions, T3—10-day follow-up) in MS and non-MS groups. Results are presented as median accompanied by 95% confidence intervals (CI) (lower; upper limit). Panel (**a1**) shows prothrombin time (PT), (**a2**)—international normalized ratio (INR), (**b**)—thrombin time (TT), and (**c**)—activated partial thromboplastin time (aPTT).

In both groups, a statistically significant prolongation of PT was observed immediately after the 10-session WBC protocol. The increase was greater in the non-MS group (Δ Changes: *p* < 0.001, d = 0.99) compared with the MS group (Δ Changes: *p* = 0.004, d = 0.43). Interestingly, at the 10-day follow-up, PT values tended to return towards baseline in the non-MS group (Δ Follow-up: *p* = 0.047, d = 1.64), whereas in the MS group PT continued to rise (Δ Follow up; *p* = 0.008, d = 0.16). INR followed a similar pattern, with a significant increase observed at T2 in both the non-MS group (Δ Changes: *p* < 0.001, d = 0.99) and the MS group (Δ Changes: *p* = 0.005, d = 0.67). During follow-up, INR remained elevated in the MS group (Δ Follow-up: *p* = 0.8, d = 1.57), while in the non-MS group the effect was attenuated but still statistically significant (Δ Follow up: *p* = 0.047, d = 1.64). Changes in TT differed between groups. In the non-MS group, a consistent decrease was observed both immediately after WBC (T2) (Δ Changes: *p* = 0.008, d = 0.42) and at T3 (Δ Changes: *p* = 0.062, d = 0.57). In contrast, the MS group showed a slight reduction at T2 (Δ Changes: *p* = 0.005), d = 0.53), which was followed by an increase above baseline values at T3 (Δ Follow-up: *p* = 0.050, d = 0.58). No statistically significant changes were detected in aPTT at any time point in either group. Median values remained stable, with only minor non-significant fluctuations. All statistically significant findings were accompanied by moderate to large effect sizes (Cohen’s d), with the strongest effects observed for INR at follow-up in both the MS (d = 1.57) and the non-MS groups (d = 1.64). Changes in PT, TT and aPTT in MS and non-MS groups across the three study time points are presented in Figure 5.

An additional analysis was performed in smaller, age-matched subgroups (MS *n* = 21, non-MS *n* = 21) to assess whether the age difference between groups influenced the observed results. Due to the limited number of participants available at T3, this analysis was restricted to changes between T1 and T2 only. The direction of changes in coagulation parameters in the age-matched analysis remained consistent with those observed in the full study population (Table 4).

In the non-MS subgroup, PT and INR continued to increase significantly at T2 compared with baseline. In contrast, in the MS subgroup the magnitude of these changes was smaller and did not reach statistical significance. Effect size estimates (Cohen’s d) ranged from small to large. This may indicate that despite the loss of statistical significance in some parameters, the magnitude of the changes remained comparable to those observed in the full cohort.

### 3.3. Measurements of CFF Parameters Following WBC in MS and Non-MS Participants

The CFF assay revealed no significant changes in clot formation kinetics or fibrinolytic efficiency in response to WBC in either group. Although minor fluctuations were observed across V_maxC_, V_maxF_, and A_max_, these shifts were small in magnitude and did not reach statistical significance (Table 5).

## 4. Discussion

The lack of fully effective pharmacological treatment for MS has prompted heightened interest in minimally invasive, non-pharmacological therapies. WBC is increasingly recognized as a supportive therapy in neurological rehabilitation. Although its clinical use continues to expand many aspects of its systemic effects, including its effects on hemostasis, remain largely unexplored. This issue is particularly relevant in patients with MS (especially those with active relapsing–remitting disease) who exhibit increased activity of coagulation factors (e.g., prothrombin and factor X) and enhanced thrombin generation compared with healthy controls [26]. Given these observations, an important question is whether repeated exposure induces measurable changes in hemostasis and whether these effects differ between individuals with and without MS.

To our knowledge, this is the first study to assess thrombotic potential using a multimodal approach, including plasma coagulation testing, assessment of semi-physiological thrombus formation under flow conditions (T-TAS^®^), and kinetic analysis of clot formation and fibrinolysis (CFF assay) in patients with MS before and after WBC therapy. This integrative methodology allowed us to capture clot initiation, propagation, fibrin stabilization, and lysis, which are key phases relevant to thrombotic risk in the context of WBC.

T-TAS was first introduced in 2011 as a microchip-based system designed to assess thrombus formation under flow conditions and it offers the possibility of assessing the potential risk of bleeding or thrombosis. Since its development the use of T-TAS has increased markedly across a variety of clinical settings, including cardiovascular diseases, bleeding disorders, and the monitoring of antithrombotic therapies [27]. Its applications include studies on anticoagulant and antiplatelet therapies, atrial fibrillation, and coronary artery disease [25]. It has been demonstrated that T-TAS has the capacity to detect changes in whole-blood thrombogenicity in circumstances in which conventional coagulation assays may not reveal significant abnormalities [25]. However, its application in neurological populations remains limited, with a notable lack of studies investigating thrombus formation dynamics in patients with MS. Data from cardiovascular populations indicate that T-TAS is sensitive to acute changes in blood thrombogenicity. For instance, significantly higher AR-AUC values have been reported in patients with acute myocardial infarction compared to those with stable coronary artery disease, with median values of approximately 1770 vs. 1670, respectively, and a further decrease observed in the chronic phase [28]. This finding indicates that T-TAS may primarily reflect dynamic, state-dependent changes in thrombogenicity.

Given that conditions such as deep vein thrombosis and recent embolism (<6 months) are recognized contraindications to WBC [29], a thorough understanding of the hemostatic response to extreme cold exposure is particularly important in certain patient populations, including individuals with MS.

Although changes in AUC did not reach statistical significance, different response trajectories were observed between the groups. In the non-MS group, AUC showed an initial decrease followed by a subsequent increase, whereas the MS group exhibited a more unidirectional pattern over time. The absence of significant alterations in this study may indicate preserved hemostatic balance and the absence of a notable prothrombotic shift following WBC.

The findings of this study indicate that WBC could modulate individual components of the coagulation system, with notable differences in response trajectories between MS and non-MS participants.

The changes observed in PT and TT suggest that WBC primarily modulated the extrinsic and terminal phases of the coagulation cascade, while the intrinsic pathway (aPTT) remained unaffected. Because PT reflects the function of the extrinsic and common coagulation pathways, particularly factors II, V, VII and X [30], the consistent prolongation of PT in both groups may indicate a transient decrease in extrinsic pathway activity. Importantly, this effect was modest and remained within physiological limits. It is worth noting that, in MS, the results of conventional plasma clotting time tests, including PT, are inconsistent in the literature and depend on the population and disease stage [31], which limits the ability to unequivocally link the observed short-term changes in PT to a specific mechanism. Although derived from a distinct clinical context, evidence from neurological studies suggests that dynamic changes in PT may reflect transient modulation of the hemostatic system. For instance, in patients with acute ischemic stroke treated with intravenous thrombolysis, an increase in PT over time was associated with unfavorable three-month outcomes. This suggests that even minor shifts in this parameter may capture subtle alterations in coagulation balance [32].

Furthermore, TT exhibited a group-dependent pattern: both cohorts showed an acute decrease immediately after WBC, suggesting transient facilitation of fibrin formation, followed by divergent long-term responses. In non-MS individuals, TT returned to values slightly below baseline, whereas in MS participants it rebounded above baseline at follow-up, indicating a potential adaptive re-equilibration of fibrin polymerization processes (Table 3, Figure 5). This biphasic response observed in MS may reflect a delayed normalization or an adaptive rebalancing of fibrin polymerization rather than a sustained procoagulant effect. As TT reflects the efficiency of fibrinogen conversion into fibrin, the observed changes may indicate functional modulation at the level of fibrin formation.

In this study the intrinsic pathway (aPTT) remained unchanged, supporting the specificity and mild nature of these effects.

Notably, previous studies investigating WBC in MS or other populations have focused primarily on markers such as plasma fibrinogen concentration and platelet count providing information on the presence of substrates of fibrin degradation, but not on the kinetics and quality of the clotting process. This approach is consistent with findings from previous studies, in which elevated fibrinogen levels have been demonstrated to correlate with disease severity [33]. In one study WBC was associated with a statistically significant increase in fibrinogen levels among healthy women, with an average rise of 34.13% observed following a 10-session protocol. In MS patients, a similar trend was noted (44% increase); however, the change was not statistically significant. Notably, baseline fibrinogen levels did not differ significantly between the groups [34]. Moreover, a study in patients with rheumatologic diseases has reported minimal or no significant changes in conventional hemostatic markers. Women with rheumatoid arthritis undergoing regular WBC showed no alteration in plasma fibrinogen levels or viscosity [35], suggesting that the intervention did not adversely affect these basic hemorheological indices. Interestingly, there is some evidence that WBC might reduce fibrinolytic activity in chronic inflammatory conditions such as rheumatoid arthritis [22]. However, these findings are not yet conclusive and may depend on the patient population and baseline characteristics. This study complements previous findings by offering a more functional perspective on coagulation, which was not addressed in existing research.

Despite the paucity of data on dynamic assays such as CFF in MS, the influence of fibrin clot structure on fibrinolysis is well established. Denser fibrin networks exhibit reduced permeability and increased resistance to lysis, indicative of a prothrombotic phenotype. Similar alterations have been reported in a variety of neurological conditions, including ischemic stroke and Parkinson’s disease [36,37]. In this context, CFF offers a dynamic assessment of clot formation and breakdown in patients with MS.

In the present study, no statistically significant alterations were detected in any of the CFF parameters following WBC, in either the MS or non-MS group. These findings may suggest that WBC does not substantially alter the structural stability or breakdown potential of the fibrin clot, indicating that the intervention may primarily affect early-phase coagulation processes, as reflected in PT and TT. Notably, the absence of measurable disturbances in fibrin polymerization dynamics or clot lysis capacity suggests that repeated WBC exposure may not impair the coordinated balance between coagulation and fibrinolysis. This further underscores the value of assessing dynamic hemostatic parameters in neurological populations, especially when evaluating the safety of therapies such as WBC.

Hemostatic alterations in MS are increasingly recognized as active contributors to disease mechanisms and have been linked to disease activity and symptomatology. Fibrinogen crossing a disrupted blood–brain barrier and depositing in MS lesions can activate microglia and augment neuroinflammation [34]. Clinical studies have reported that higher plasma fibrinogen levels tend to coincide with relapse or MRI-active disease [38] underscoring fibrinogen’s role as an inflammation-linked biomarker. In addition, elevated factor XII activity in MS was associated with more frequent relapses in one study, suggesting that coagulation factors might even drive disease activity [26]. Moreover, patients with MS show signs of heightened platelet activation (e.g., increased P-selectin expression and circulating platelet microparticles) compared with controls [39]. Such prothrombotic and hypofibrinolytic shifts may contribute not only to CNS pathology but also to systemic complications.

From a clinical perspective, even subtle modulation of coagulation in MS patients be clinically relevant, given the increasing recognition of thrombotic risk and involvement in MS pathophysiology. As WBC is increasingly used as part of neurorehabilitation protocols, confirming its hemostatic safety is essential, particularly in patients with chronic inflammation and altered endothelial function, which are prevalent in MS. These preliminary findings may support the hemostatic safety of repeated WBC in appropriately screened patients without significant vascular comorbidities. However, in patients with MS and coexisting vascular disease, eligibility for WBC should be determined on an individual basis, taking into account cardiovascular, cerebrovascular and thromboembolic history, current antithrombotic therapy, overall clinical status, and the individual risk–benefit profile. Further research is needed to explore the potential relationship between hemostasis and clinical outcomes.

Nonetheless, the clinical implications of repeated or long-term WBC exposure in patients with MS require further investigation, particularly in those with coexisting vascular risk factors.

### Limitations

The interpretation of the T3 findings should be considered in the light of the smaller sample size available at this time point and the relatively short, 10-day follow-up period which limited the assessment of delayed or persistent changes in hemostatic function could not be assessed. Future studies with longer follow-up and additional assessment time points would help to further characterize the temporal effects of repeated WBC on hemostatic parameters. Another limitation was the age difference between the study groups. Participants with MS were younger, whereas the non-MS group consisted predominantly of older adults with osteoarthritis. Ageing is associated with a more prothrombotic hemostatic profile due to endothelial dysfunction, enhanced platelet activation, changes in coagulation factors, and fibrin clot structure, including reduced susceptibility to fibrinolysis [40]. Therefore, age may have influenced the observed between-group differences. The study groups also differed in sample size, with fewer participants in the MS group. These discrepancies reflect the clinical reality of patient recruitment, where older patients with MS often did not meet eligibility criteria for WBC due to contraindications or reduced functional capacity, while WBC is more commonly prescribed in older patients with degenerative joint or spine disorders. To address these issues, an additional comparison was conducted in smaller age-matched subgroups, which confirmed that the direction of the observed changes remained consistent, suggesting these differences were unlikely to fully account for the findings.

An influence of ongoing DMT treatment on baseline hemostatic measurements and, consequently, on the interpretation of the observed response patterns cannot be completely excluded. Rare hematological adverse events with potential relevance to hemostasis, including thrombocytopenia and thrombotic microangiopathy, have been reported with some DMTs [41]. Moreover, experimental studies suggest that dimethyl fumarate and glatiramer acetate may inhibit platelet activation [42,43]. However, these findings were obtained in vitro using human platelets and in animal models and therefore do not establish clinically meaningful effects on routine coagulation parameters in patients with MS.

Finally, the exploratory, nonrandomized, and uncontrolled study design, including the absence of a sham-treated or no-WBC control group, limits causal interpretation. Accordingly, the observed changes should be interpreted as preliminary, hypothesis-generating findings.

## 5. Conclusions

This study suggests that repeated WBC sessions exert only minor, pathway-specific effects on plasma coagulation, without compromising the overall hemostatic balance. The absence of procoagulant activation, together with the subtle modulation of selected clotting times, indicates that WBC was not associated with clinically relevant disturbances in hemostasis in patients with MS. No adverse events or abnormal coagulation findings were reported among participants during or after the intervention. The findings of this study support the short-term safety of WBC in the context of hemostatic function. However, given the reduced follow-up sample size, these findings should be interpreted cautiously and cannot confirm the long-term hemostatic safety of WBC. Future studies should include larger cohorts and longer follow-up periods to determine whether the observed tendencies represent transient adaptive changes or persist over time.

## Figures and Tables

**Table 1:** The baseline demographics and clinical characteristics of the study participants.

	All (*n* = 72)	Non-MS (*n* = 43)	MS (*n* = 29)
	**Mean ± SD** **(95%CI)**		
**Age** (year)	54.36 ± 14.31 (50.99; 57.72)	61.59 ± 9.52 * (58.63; 64.56)	43.90 ± 3.94 (38.59; 49.21)
**Weight** (kg)	69.66 ± 12.84 (66.66; 72.65)	69.07 ± 12.26 (65.30; 72.84)	70.79 ± 13.50 (65.65; 75.93)
**Height** (cm)	166.92 ± 8.56 (164.92; 168.91)	164.81 ± 7.78 * (162.42; 167.21)	170.00 ± 8.98 (166.58; 173.42)
**No. WBC in the past**	5.32 ± 6.93 (3.69; 6.95)	5.64 ± 5.04 * (4.07; 7.21)	4.97 ± 9.17 (1.49; 8.45)
	**N (%)**		
**Gender**			
Females	62 (83.78)	39 (88.64)	22 (75.86)
Males	11 (14.86)	4 (9.09)	7 (24.14)
**Education**			
Primary education	2 (2.70)	2 (4.54)	0
Secondary education	28 (37.84)	21 (47.73) *	7 (24.14)
Higher education	43 (58.11)	20 (45.45) *	22 (75.86)
**Working status**			
Employed	45 (60.81)	22 (50.00) *	22 (75.86)
Unemployed	1 (1.35)	0	1 (3.45)
Retiree	23 (31.08)	19 (43.18) *	4 (13.79)
Disability pensioner	4 (5.40)	2 (4.54)	2 (6.90)
**Comorbidities**			
No	36 (48.65)	18 (40.91)	17 (58.62)
Yes	37 (51.35)	25 (59.09)	12 (41.38)
**Smoking**			
No	61 (82.43)	36 (81.82)	24 (82.76)
Yes	12 (16.22)	7 (18.18)	5 (17.24)
**Special diet**			
No	64 (86.49)	34 (77.27) *	29 (100.00)
Yes	9 (12.16)	9 (20.45)	0
**Physical activity**			
More than 2 times per week	33 (44.59)	20 (45.45)	12 (41.38)
2 times per week	15 (20.27)	11 (25.00)	4 (13.79)
1 time per week	13 (17.57)	7 (15.91)	6 (20.69)
1 time per month	3 (4.05)	1 (2.27)	2 (6.90)
No physical activity	9 (12.16)	4 (9.09)	5 (17.24)
**WBC in the past**			
No	18 (24.32)	3 (6.82) *	15 (50.00)
Yes	55 (74.32)	40 (90.91) *	15 (50.00)

* *p* < 0.05.

**Table 2:** Comparison of changes in AUC parameters between non-MS and MS groups was performed using the T-TAS^®^ system across two-time intervals (Δ Changes: T2–T1; Δ Follow-up: T3–T1). Statistical analysis was conducted using the Wilcoxon signed-rank test. Results are expressed as median accompanied by 95% confidence intervals (CI) (lower; upper limit). Effect sizes were calculated using Cohen’s d, with values ≥ 0.8 and *p* < 0.05 interpreted as indicating a strong clinical effect.

Groups:	Non-MS						MS					
Variables	Δ Changes (T2–T1)	d	*p*	Δ Follow-Up (T3–T1)	d	*p*	Δ Changes (T2–T1)	d	*p*	Δ Follow-Up (T3–T1)	d	*p*
**T-TAS** [AUC]	−31.1 (−81.28; 26.91)	0.20	0.133	10.80 (−85.33; 158.9)	0.61	0.463	−8.80 (−54.31; 39.12)	0.05	0.755	−23.50 (−42.48; 94.42)	1.22	0.499

**Table 3:** Comparison of changes in coagulation parameters (PT, TT and aPTT) between non-MS and MS groups at different time points (T2–T1 and T3–T1), with effect sizes (Cohen’s d) and statistical significance (*p*-values). Statistical analysis was performed using the Wilcoxon signed-rank test. Results are presented as median (95% CI: lower limit; upper limit). Results highlighted in bold indicate statistically significant results (with *p* < 0.05).

Groups:	Non-MS						MS					
Variables	Δ Changes (T2–T1)	d	*p*	Δ Follow-Up (T3–T1)	d	*p*	Δ Changes (T2–T1)	D	*p*	Δ Follow-Up (T3–T1)	d	*p*
**PT [sec]**	2.2 (1.92; 2.98)	0.99	**<0.001**	0,73 (0.99; 2.63)	1.64	**0.047**	1.05 (1.35; 2.29)	0.43	**0.004**	2.9 (0.2; 0.57)	0.16	**0.008**
**INR**	0.23 (0.20; 0.31)	0.99	**<0.001**	0.06 (0.37; 0.98)	1.64	**0.047**	0.11 (0.14; 0.25)	0.67	**0.005**	0.11 (0.02; 0.06)	1.57	**0.008**
**TT [sec]**	−0.4 (1.20; 1.85)	0.42	**0.008**	−0.8 (0.76; 1.91)	0.57	0.062	−0.35 (1.09; 1.86)	0.53	**0.005**	0.95 (0.78; 2.22)	0.58	0.050
**aPTT [sec]**	0.43 (2.72; 4.27)	0.10	0.248	0.85 (2.52; 6.33)	0.19	0.328	0.25 (2.14; 3.69)	0.09	0.597	−0.55 (1.31; 4.03)	0.11	0.401

**Table 4:** Comparison of changes in coagulation parameters (PT, TT and aPTT) between non-MS and MS groups in age-matched subgroups, with effect sizes (Cohen’s d) and statistical significance (*p*-values). Results highlighted in bold indicate statistically significant results (with *p* < 0.05).

Groups	Non-MS			MS		
Variables	Δ Changes (T2–T1)	d	*p*	Δ Changes (T2–T1)	d	*p*
PT [sec]	2.1 (0.44; 2.69)	0.93	**0.025**	0.75 (−0.29; 1.36)	0.43	0.073
INR	0.21 (0.04; 0.28)	0.70	**0.021**	0.08 (−0.03; 0.14)	0.42	0.076
TT [sec]	0.00 (−0.67; 0.31)	0.12	0.526	−0.45 (−1.65; −0.21)	0.53	**0.035**
aPTT [sec]	0.65 (−0.64; 2.47)	0.31	0.316	0.25 (−0.99; 1.45)	0.06	0.643

**Table 5:** Comparison of changes in CFF parameters (V_maxC_, V_maxF_ and A_max_) between non-MS and MS groups at different time points (T2–T1 and T3–T1), with effect sizes (Cohen’s d) and statistical significance (*p*-values). Results highlighted in bold indicate statistically significant results (with *p* < 0.05).

Groups:	Non-MS						MS					
Variables	Δ Changes (T2–T1)	d	*p*	Δ Follow-Up (T3–T1)	d	*p*	Δ Changes (T2–T1)	d	*p*	Δ Follow-Up (T3–T1)	d	*p*
**V_max Clotting_**	−0.02 (−0.06; 0.01)	0.26	**0.043**	0.01 (−0.12; 0.19)	1.01	0.98	−0.01 (−0.06; 0.02)	0.13	0.372	0.00 (−0.03; 0.06)	0.39	0.320
**V_max Fibrinolysis_**	−0.02 (−0.04; 0.01)	0.16	0.221	0.03 (−0.02; 0.06)	1.19	0.412	0,01 (−0.07; 0.04)	0.13	0.810	0.00 (−0.03; 0.06)	0.25	0.8203
**A_max_**	−0.02 (−0.09; 0.06)	0.15	0.617	0.11 (−0.04; 0.25)	0.99	0.129	0.02 (−0.09; 0.07)	0.04	0.861	−0.07 (−0.16; 0.03)	0.54	0.163

## Data Availability

The datasets generated during and/or analyzed during the current study are available from the corresponding author on reasonable request.
